# Antimicrobial Resistance Determinants in Genomes and Plasmids from *Acinetobacter baumannii* Clinical Isolates

**DOI:** 10.3390/antibiotics10070753

**Published:** 2021-06-22

**Authors:** Itziar Chapartegui-González, María Lázaro-Díez, Santiago Redondo-Salvo, Jesús Navas, José Ramos-Vivas

**Affiliations:** 1 Department of Microbiology and Immunology, UTMB, Galveston, TX 77550, USA; 2 Instituto de Agrobiotecnología, CSIC-Gobierno de Navarra, 31192 Mutilva, Spain; marialazarodiez@gmail.com; 3 Instituto de Biomedicina y Biotecnología de Cantabria (IBBTEC), Universidad de Cantabria-CSIC, 39011 Santander, Spain; santiago_redondo@yahoo.es; 4 Grupo BIOMEDAGE, Facultad de Medicina, Universidad de Cantabria, 39011 Santander, Spain; navasj@unican.es; 5 Instituto de Investigación Valdecilla (IDIVAL), 39011 Santander, Spain; 6 Research Group on Foods, Nutritional Biochemistry and Health, Universidad Europea del Atlántico, 39011 Santander, Spain; jose.ramos@uneatlantico.es; 7 Universidad Internacional Iberoamericana, Campeche 24560, Mexico

**Keywords:** *Acinetobacter baumannii*, ESKAPE pathogens, antimicrobial determinants, plasmids, bioinformatics, WGS, Illumina

## Abstract

*Acinetobacter baumannii* is a Gram-negative coccoid rod species, clinically relevant as a human pathogen, included in the ESKAPE group. Carbapenem-resistant *A. baumannii* (CRAB) are considered by the World Health Organization (WHO) as a critical priority pathogen for the research and development of new antibiotics. Some of the most relevant features of this pathogen are its intrinsic multidrug resistance and its ability to acquire rapid and effective new resistant determinants against last-resort clinical antibiotics, mostly from other ESKAPE species. The presence of plasmids and mobile genetic elements in their genomes contributes to the acquisition of new antimicrobial resistance determinants. However, although *A. baumannii* has arisen as an important human pathogen, information about these elements is still not well understood. Current genomic analysis availability has increased our ability to understand the microevolution of bacterial pathogens, including point mutations, genetic dissemination, genomic stability, and pan- and core-genome compositions. In this work, we deeply studied the genomes of four clinical strains from our hospital, and the reference strain ATCC^®^19606^TM^, which have shown a remarkable ability to survive and maintain their effective capacity when subjected to long-term stress conditions. With that, our aim was presenting a detailed analysis of their genomes, including antibiotic resistance determinants and plasmid composition.

## 1. Introduction

The discovery of penicillin in 1928 marked the beginning of the antibiotic era, which meant a revolution in public health. However, their wrong utilization in the last decades has significantly increased the number of antimicrobial-resistant strains [1]. Despite the fact that multidrug-resistant bacteria pose a threat to both global morbidity and mortality, pharmaceutics research and development has not been able to meet the clinical need for new antibiotics. As a consequence, the World Health Organization (WHO) has created “a priority list of antibiotic-resistant bacteria”, in which the *Acinetobacter* genus is included [2]. *Acinetobacter* spp. are Gram-negative coccoid rods, mostly of an environmental origin, but with some species being clinically relevant as human pathogens, especially *A. baumannii*, a human pathogenic one included in the ESKAPE group [3]. Importantly, carbapenem-resistant *A. baumannii* (CRAB) strains are considered by the WHO as critical priority pathogens for the research and development of new antibiotics due to their limiting therapeutic options [2]. *A. baumannii* is usually described as a low virulence pathogen, with its lethality being associated with the activation of the immune system due to its highly toxic lipopolysaccharide (LPS) [4,5]. As a human opportunistic pathogen, it is usually associated with the most critical patients in Intensive Care Units (ICUs) in whom they could cause bacteremia, pneumonia, and different wound infections, among other things [6,7].

Some of the most important features of this pathogen are being naturally antimicrobial-resistant, and being able to acquire rapid and effective new resistant determinants against last-resort clinical antibiotics [4], mostly from *Pseudomonas*, *Salmonella*, or *Escherichia* strains [6]. Therefore, antibiotic resistance genes should be closely surveilled in *Acinetobacter* strains isolated at the clinical setting. For a long time, their natural antibiotic resistance was considered a consequence of the low wall permeability, but now it is known that antimicrobial efflux pumps are crucial for this phenotype [8,9]. As the definition of multidrug-resistant (MDR) in practice depends on the genus, for *Acinetobacter,* the following terms have been suggested [10]: (i) MDR, resistant to three different antimicrobial classes; (ii) XDR (extensively drug-resistant), resistant to more than one compound in all except two antimicrobial classes (MDR resistant to carbapenem); (iii) PDR (pandrug-resistant), resistant to all compounds in every antimicrobial class (XDR resistant to polymyxin and tigecycline). Notably, PDR strains from diverse sources have already been described [11,12,13]. *A. baumannii* strains display the main antimicrobial-resistant mechanisms described for other Gram-negative bacteria. Beta-lactam resistance in *Acinetobacter* species is associated with a broad repertoire of β-lactamases, which confer resistance against penicillin, cephalosporins, and carbapenems. Within this group, the most common in *A. baumannii* are oxacillinases (OXAs), with more than 400 different enzymes clustered in six homology groups with different targets [5]: OXA-51, chromosomic and intrinsic, the biggest subgroup; OXA-23, chromosomic or plasmid; OXA-40/24, acquired carbapenemases; and OXA-58, OXA-143, and OXA-48, more recently described as having low hydrolytic activity. The presence of plasmids and other mobile genetic elements in *A. baumannii* genomes contributes to its ability to acquire new resistance mechanisms rapidly [14]. Moreover, a replicon-based typing method for *A. baumannii* resistance plasmids has been recently proposed [15]. However, although *A*. *baumannii* has arisen as an important human pathogen, information about these genetic elements is still not well understood.

Nowadays, genomic analysis from whole-genome sequencing (WGS) has increased our ability to evaluate bacterial pathogens’ microevolution, which includes point mutations, antimicrobial-resistant determinant dissemination, genomic stability across the world, and both pan- and core-genome compositions [16]. These new tools, as well as genetic manipulation, have allowed us to gain a better comprehension of some of these pathogens’ features, such as host–pathogen interaction mechanisms, their virulence factors, and their environmental survival ability [17,18,19]. The presence or absence of classic virulence factors, evidenced by robust animal or cellular models, is of special interest due to the link to its pathogenicity with the activation of the host’s immune system [20,21]. In this species, the presence of iron uptake mechanisms is remarkable and is related to its survival ability, acinetobactin being the most common and studied system whose presence is strain-specific [19].

Four *A. baumannii* clinical strains isolated in our hospital and included in this study showed a remarkable ability to survive and maintain their effective capacity when subjected to stress conditions, such as those found by nosocomial bacteria in the hospital conditions, despite their lack of virulence in cell models, which is the key point for their transmission [22,23]. Considering the above, the aim of this work was to present a detailed analysis of plasmids and antibiotic resistance determinants.

## 2. Results

### 2.1. Genomic Annotations and Subsystem Categories

Genome sizes and % GC, subsystem and predicted protein-coding sequences (CDS) by RAST and Prokka annotations, and the sequence typing are shown in Table 1. Genome sizes range from 3.81 Mb to 4.06 Mb, all of them with a GC content of around 39% (±0.1). The RAST annotation yielded between 3556 and 3882 protein-coding sequences, belonging to between 451 and 462 subsystems, while Prokka annotation predicted between 3542 and 3886 CDS.

**Figure 1:**
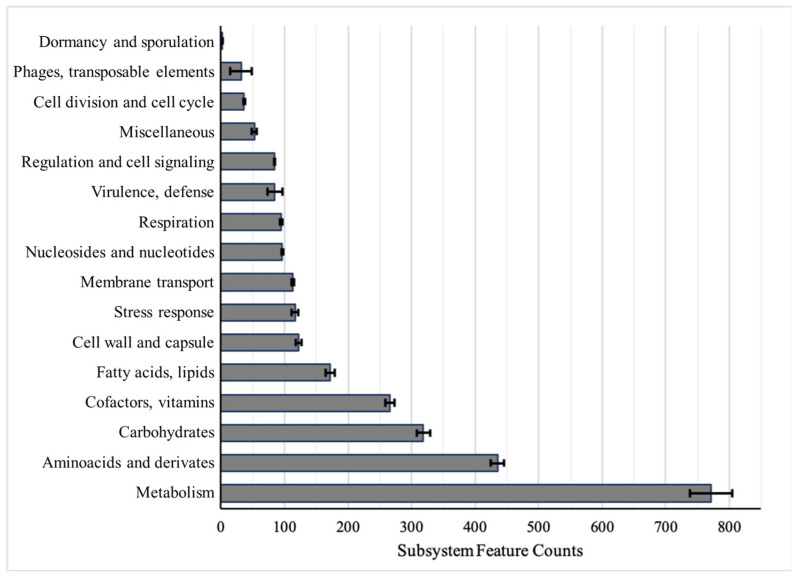
Protein-coding sequences included in each subsystem category. Each bar shows the average of five strains ± SE (standard error).

The average of the CDS included in each subsystem is shown in Figure 1. Most belonged to metabolism and molecular compounds such as amino acids and carbohydrates, among other things. Between 75 and 96 CDS fit into the “Virulence and defense” subsystem category (mean = 84.6 ± 11.47 standard deviation -SD-), where antimicrobial resistance-related genes are included.

### 2.2. Strain Comparison and Pan-Genome Analysis

**Figure 2:**
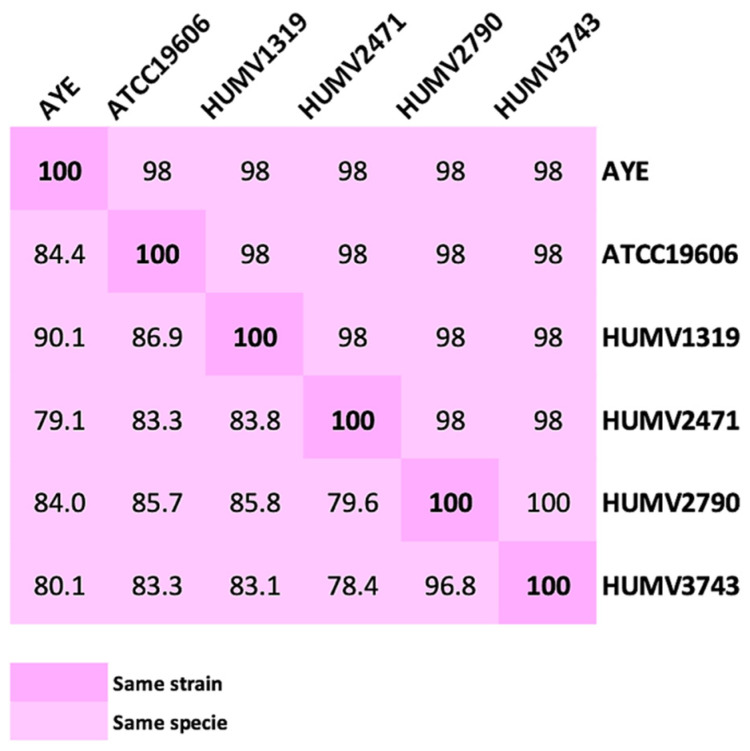
Matrix distance values among *A. baumannii* strains. The upper part shows ANI values; the lower part, DDH values. An AYE reference strain was also included to confirm the bacterial species.

A matrix created using Average Nucleotide Identity (ANI) and DNA-DNA hybridation (DDH) values shows the relationship and similarities among our *A. baumannii* and reference strains (ATCC^®^19606^TM^ and AYE) (Figure 2). Both methods indicated that HUMV2790 and HUMV3743 strains were the most similar (100 and 96.8% as ANI and DDH values, respectively), while HUMV1319 and HUMV3743 were the most far among them according to DDH value (83.1%).

**Figure 3:**
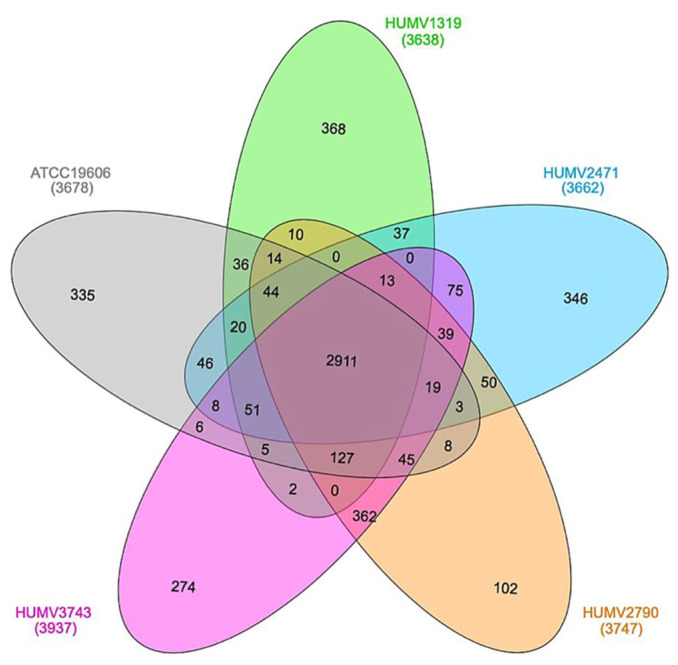
Venn diagram with pan-genome composition of five *A. baumannii* strains. In the middle, core-genome size. Each value corresponds with the number of CDS shared for two or more strains, and the single ones in the end of each ellipse.

The five strains’ pan-genome was composed of 5244 genes, while the core-genome was composed of 2911 genes, the former being the total number of different CDS and the latter being the number of them shared among all the group. Figure 3 shows the relationship among the isolates, with the numbers signifying the common CDS for each couple or group. Again, the strains HUMV2790 and HUMV3743 are the couple that shares the largest number of genes (362), with the lowest being between HUMV1319 and HUMV3743 (2).

### 2.3. Susceptibility of A. baumannii to Antimicrobial Agents

The MICs for the seven compounds tested are shown in Table 2. Strains HUMV2790 and HUMV3743, isolated from skin ulcer and wound exudate, respectively, were resistant to the all the tested compounds. The five strains were resistant to ampicillin, meropenem, and erythromycin.

### 2.4. Antimicrobial Resistance Determinants

In Table 3, the antimicrobial resistance genes detected for each strain are shown and related to their resistance to aminoglycosides, β-lactams, and sulfonamides.

All strains carried at least one aminoglycoside modifying enzyme (AME), the type and number of them among the isolates being variable. The reference strain was the only one susceptible to gentamicin, which only harbors one AME encoding gene (*ant(3’’)-IIa*). The strains HUMV1319 and HUMV3743 showed the biggest repertoire of AME genes, with representation of the three enzymatic classes: acetyltransferases (AAC), nucleotidyltransferases (ANT), and phosphotransferases (APH). Related to β-lactam compound resistance, all the strains carried two encoding genes for the chromosomic endogenous class D β-lactamase OXA-51, with different allotypes and an *Acinetobacter*-derived cephalosporinase (ADC). The strains HUMV1319 and HUMV2471 also presented a commonly plasmid-harbored *bla_OXA-24/40_* gene. Additionally, three out the five strains carried the gene sul, related to sulfonamide resistance.

All the clinical samples were resistant to ciprofloxacin (Table 2). In all of them, point mutations in quinolone resistance-determining regions (QRDR) of *gyrA* and *parC* genes were detected. In the *gyrA* gene, the amino acid substitution Ser-81→Leu-81 was common for all the strains. However, in the *parC* product, the Ser-84→Leu-84 mutation was present in all strains except HUMV2471, which showed a replacement in Glu-88→Lys-88. *A. baumannii* numbering was used in both cases. 

Related to colistin resistance, mutations in *lpxACD* and *pmrAB* were mapped. The LpxA subunit showed no variations among the strains; LpxC showed a substitution Asp-287 → Asn-287 in ATCC^®^19606^TM^, while in LpxD, different strains harbored different mutations: in HUMV2471, Val-63 → Ile-63; in HUMV1319, Glu-73 → Asp-73; and in HUMV2790 and HUMV3743 (both colistin-resistant), Glu-117 → Lys-117. In regard to the *pmrAB* operon, in position 119 of PmrA, the HUMV1319 presented a Thr while the others had a Ser. The 4 clinical strains harbored His-440 in PmrB instead of the Asn-400 of the reference strain ATCC^®^19606^TM^. The plasmid-borne gene *mcr-1*, also related to colistin resistance, was not detected in any strain.

All the strains harbored a broad repertoire of efflux pumps genes, including resistance-nodulation-cell division (RND) multidrug efflux pumps (AdeABC, AdeFGH, and AdeIJK), small multidrug resistance (SMR) (AbeS), and multidrug and toxic extrusion (MATE) (AbeM) family efflux pumps. Regulatory genes *adeL* and *adeN* were also identified in all the strains, related to *adeFGH* and *adeIJK* overexpression respectively, as well as the *adeRS* operon (*adeABC*), except HUMV2471, which only presented the *adeR* subunit.

### 2.5. Plasmid Prediction and Analysis

**Figure 4:**
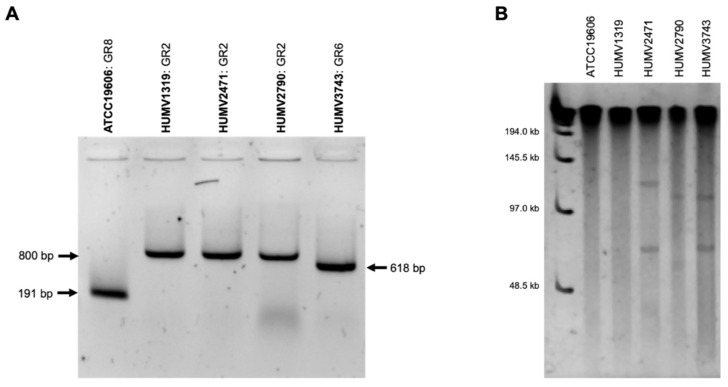
Molecular detection of plasmids in *A. baumannii*. (**A**) PCR-based replicon type (PBRT) and (**B**) PFGE gel for plasmids detection in *A. baumannii* strains. In (**B**), a molecular size ladder (Lambda ladder PFGE marker, NEB Biolabs) was used in first lane.

Using Bertini et al. [15] specific resistance plasmid primers, positive results (Figure 4A) were obtained for all the strains in three different typing groups (GR): ATCC^®^19606^TM^ GR8; HUMV1319, HUMV2471, and HUMV2790 GR2; and HUMV3743 GR6.

Through a PFGE electrophoresis after an S1 digestion plasmid, the presence of plasmids was obtained for HUMV2471, HUMV2790, and HUMV3743 strains, with sizes ranging from 48.5 to 145.5 kb (ladder) (Figure 4B).

The PLACNETw server predicted putative plasmids in all *A. baumannii* Illumina draft genomes analyzed (Figure S1): in ATCC^®^19606^TM^, two putative small plasmids of 11 and 15 kb; in HUMV1319, a small 8.90 kb plasmid and three smaller accessory structures from 1 to 7 kb with not enough identity to be considered as plasmids; in HUMV2471, two big plasmids of 118.39 and 69.2 kb and two small ones of 6 and 8.95 kb; in HUMV2790, a 105.1 kb big plasmid and an 11.55 kb small one; and in HUMV3743, two big plasmids of over 107 kb and 78.73 kb.

The 15 kb plasmid of strain ATCC^®^19606^TM^ harbored a MOB_Q_ class relaxase (detected by PLACNETw). This is a mobilizable plasmid and no genes coding for a Type IV Secretion System have been detected. Plasmid *pMAC* (NCBI: NC_006877) is the most similar plasmid with a 99.97% ANI.

The 8.90 kb plasmid of strain HUMV1319, the 8.95 kb plasmid of HUMV2471, and the 11.55 kb plasmid of HUMV2790 are a group of similar plasmids to plasmid *pMMCU3* (NCBI: NC_019199). The ANI is 100%, 99.87% and 99.95% respectively.

PLACNETw detected a MOB_F_ class relaxase in the 69.2 kb plasmid of strain HUMV2471 and in the 78.73 kb plasmid from HUMV3743. Moreover, both plasmids are putative conjugative plasmids as, in addition to the relaxase, they had genes coding for a Type IV Secretion System (*traA*, *traL*, *traE*, *traK*, *traB*, *traV*, *traC*, *traW*, *traU*, *traN*, *traN*, *traH*, *traG*, *trwB*, and *trwC*). Plasmid *pD72-2* (NCBI: NC_025111) is one of the most similar plasmids in NCBI with an ANI of 99.9% for both plasmids.

According to PLACNETw, plasmid *pABUH5-114* (NCBI: NZ_AYOI01000002) is a similar structure to the 107 kb plasmid of strain HUMV3743 and the 105.1 kb plasmid of HUMV2790. However, even with both being large plasmids, no known relaxase nor replicon was found in either plasmid. The case is similar for the 118.39 kb plasmid of strain HUMV2471, but the reference plasmid is *pABTJ2* (NCBI: NC_020524) with a 99.52% ANI. For the 6 kb plasmid of strain HUMV2471, the reference plasmid is *pRAY* (NCBI: NC_025068) with a 100% ANI.

### 2.6. Virulence Factors

Gene clusters related to siderophores (acinetobactin, actinoferrin) and aryl polyene (berninamycin) detected using antiSMASH were common in all strains and others, as K53 capsular polysaccharide (HUMV2790 and HUMV3743) was only present in some isolates. Analysis of virulence genes in the strains used in this study revealed the existence of a few classical virulence genes in all of them. The virulence of the strains would be related only to genes responsible for the synthesis of lipopolysaccharide and structural genes for the formation of the capsule. These virulence genes are listed in Table S1.

## 3. Discussion

We performed a bioinformatic analysis of five draft genomes from four *A. baumannii* clinical isolates from a Spanish hospital, and a reference strain showing a high level of resistance to most antimicrobials tested. These strains have been previously studied and do not show virulence in vitro [22]. The data from the genomic analysis agree with these results, revealing only a few genes classically implicated in the virulence of other Gram-negative pathogens (Table S1). On the other hand, some of them have a marked resistance to environmental conditions, which makes them very persistent in hospital settings [23]. This is consistent with the presence of capsular polysaccharides, which may play a relevant role in such survival under adverse environmental conditions, protecting them from external factors, including desiccation. The capsule also protects bacteria from phagocytosis, although these strains were easily captured by human neutrophils in vitro [20].

We evaluated their susceptibility to seven antibiotics. All the strains showed resistance to meropenem, ampicillin, and erythromycin. Strains also presented resistance to gentamicin, ciprofloxacin, and tetracycline. Only two strains showed resistance to the last-resort antibiotic colistin. These strains could only be considered as MDR because only one compound for each class was tested [10]. These results are in concordance with previous studies that highlight the high prevalence of antimicrobial resistance among *A*. *baumannii* strains [12,26].

The increased accessibility to WGS platforms has led to a growing number of sequenced bacterial genomes, which allows performing comparative analysis to be more comprehensive and complete. Genomic sequencing allows, among other things, identification of putative virulence factors, possible targets for diagnosis, treatment, or vaccine development, and/or identification of colonized patients in hospital outbreaks [27,28]. Comparative genomic analysis can also help in the identification of regions for the acquisition and/or spread of antimicrobial resistance, as well as the degree of variability among genome estimation [28,29].

The *Acinetobacter* genus, in particular *A. baumannii*, has aroused a lot of interest for its speed and apparent ease to acquire new antimicrobial resistance mechanisms. In this work, different databases (ResFinder and CARD) were used to identify antimicrobial resistance determinants, efflux pump encoding genes (only detected by CARD), and point mutations in target antimicrobial related genes (MEGA7, PointFinder) [30,31,32]. PointFinder was unable to determine the specific modifications related to the resistance phenotype, so they were manually mapped.

Related to aminoglycoside resistance, eight genes encoding AMEs were identified (two AAC, two ANT, and four APH), and were previously described for this bacterium [26,33,34,35]. The most prevalent gene was *ant(3”)-IIa*, common to all strains, including ATCC^®^19606^TM^, which was the only strain with gentamicin susceptibility, so these genes should be related to resistance to other aminoglycosides (Table 3).

Regarding β-lactams resistance, all strains harbored at least one cephalosporinase encoding gene (*ampC*), with a wide variety of allotypes (four enzymes in five strains) (Table 3). The most prevalent isoform was ADC-25, encoding for the closest strains HUMV2790 and HUMV3743 previously identified [36,37]. As for oxacillinase genes, all strains carried many types, the endogenous *bla_OXA-51_* being the most common with two different isoforms in each strain (six different isoforms in total). Surprisingly, ATCC^®^19606^TM^ and HUMV2471 strains harbored the same varieties, as well as HUMV2790 and HUMV3743 between them. Strains HUMV1319 and HUMV2471 carried an additional *bla_OXA-24/40_* gene, commonly plasmid-located (such as *pMMCU3*) [38]. The gene *bla_OXA-23_* was not identified in our strains, although it was the most prevalent in other studies [39,40].

Other mechanisms to promote an MDR phenotype are point mutations in target antimicrobial proteins, such as for quinolones and colistin. Quinolone targets are DNA gyrase subunit A (*gyrA*) and topoisomerase IV (*parC*), whose mutations are typically concentrated in QRDR [6,33]. Among our clinical isolates, all showed a Ser81Leu (*A*. *baumannii* numbering) substitution, the ATCC^®^19606^TM^ strain being the only one susceptible to ciprofloxacin, which harbored the serine residue at position 81. For *parC*, HUMV2471 had a Glu88Lys substitution, while the other three strains presented a Ser84Leu replacement. While *gyrA* and *parC* mutations in *A. baumannii* were described in the 1990s [41,42], a substitution in position 88 of *parC* has been more recently described [43]. Although these alterations have been widely studied, terminological differences are usually found due to the use of the *E. coli* gene sequences as the reference in protein alignments (particularly in older publications), which displaces the mutation position [26,43,44,45]. On the other hand, resistance to the last-resort antibiotic colistin has been associated with mutations in *lpxACD* operon and in *pmrAB* two components, both related to LPS biosynthesis, the pharmacological target of polymyxins [26,46]. In this study, point mutations have been found in many genes, but only one was common and exclusive for the colistin-resistant strains: Glu117Lys in *lpxD*, not previously described as far as we know. No strain harbored the *mcr-1* plasmid gene after it was manually searched for with the *tblastn* tool, traditionally linked with polymyxin resistance [47]. Moreover, all strains owned RND multidrug efflux pumps operons *adeABC*, *adeFGH*, and *adeIJK*, each one with a group of substrates, among which are β-lactams, macrolides, tetracyclines, and fluoroquinolones [33,48]. The regulatory genes *adeL* and *adeN* were also identified, which are responsible for the overexpression of *adeFGH* and *adeIJK*, respectively, and the *adeRS* operon that regulates *adeABC* expression [48,49]. Likewise, all strains were positive to other efflux pumps, the SMR *abeS* which is able to eliminate macrolides, and the MATE *abeM,* whose substrates are fluoroquinolones [33,49].

Constant and updated genomic information enables increasing knowledge about the bacterial pathogens. For these five *A. baumannii* clinical isolates, RAST software was used to classify the identified genes of the sequenced and annotated genomes in subsystems based on their function [50]. RAST was not used for the annotation of antimicrobial determinants because it usually appoints genes with enterobacterial names, although there was specific terminology for another species. The largest number of genes with identified functions were included in features associated with “*Metabolism*” (protein, nucleic, phosphorus, sulfur, nitrogenous) (Figure 1). The following, based on the number of genes, were the features related to cellular components (carbohydrates, amino acids, cofactors, lipids). Another relevant group for both pathogenic relevance and genomic representation was the “*Virulence and defense*” group, in which antimicrobial resistance genes are included, with an average value of 84.6, which represents around 2.28% of the total. This is the largest number in comparison with reference strains from the ESKAPE pathogen groups: *Enterococcus faecium* DO 1.40% (44 “*Virulence and defense*” related genes in 3124 CDS), *Staphylococcus aureus* NCTC8325 2.15% (58 in 2687), *Klebsiella pneumoniae* HS11286 1.18% (68 in 5731), *Pseudomonas aeruginosa* PAO-1 1.17% (69 in 5858), and *Enterobacter* sp. EA-1 0.97% (126 in 12,965). It should also be noted that this bacterium has a small genome compare to the others Gram-negative pathogens from this group, and hence fewer CDS.

Other studies focusing on differentiating between MDR and susceptible strains, as well as pathogenic and environmental ones, showed greater differences in the number of elements classified for metabolic functions (including catabolism and cellular processes) [17,51]. However, all strains tested here were pathogens with a multi-resistant phenotype, so these differences could not be established.

In the last decade, comparative analysis of *A. baumannii* genomes has been performed, mainly to determine pan- and core-genome sizes, which enables understanding of both the relationship and variability among strains [19,51,52,53,54]. In this work, PanOCT and InteractiVenn softwares were used to visualize not only the sizes (Figure 3), but also the relationship and number of shared genes among the five Illumina sequenced *A. baumannii* strains [55,56]. Taxonomic relationships and evolutionary distances among strains were determined using ANI [57] and DDH [58] scores including the well-known and publicly accessible genome of the multi-resistant AYE strain (#GCA_000069245.1) in order to confirm the species. Their relationship values ranged from 100 and 96.8% (ANI and DDH, respectively) for the closest strains, to 78.4% (DDH score) for the most distant ones (Figure 2). As ANI distances were determined for less restrictive values than DDH ones, they may lead to the mistake of considering two similar strains as one, so for close genomes, DDH scores are more recommended. Through the orthologs analysis, 5244 CDS for pan-genome and 2911 for core-genome were established. Accessory genes were between 102 (HUMV2790) and 368 (HUMV1319). Comparing PanOCT with ANI/DDH results, similar relationships were obtained, with HUMV2790 and HUMV3743 sharing more genes between each other (362) than among the rest. The second largest group of shared genes was 127, including all strains except HUMV2471, which also showed the lowest values in general in ANI/DDH matrix. Pan-genome and core-genome sizes in other studies vary depending on the strain set and isolate origin, being also affected by sample size, with pan-genome values between 3000 and 6500, similar to our results [52,53,54].

*Acinetobacter baumannii* human pathogenic strains are clinically relevant for their high resistance rate. Plasmids, as mobile elements, may disseminate genetic resistance determinants [59,60]. Despite this pathogen relevance, information about *A. baumannii* plasmids is scarce, with structures ranging from 2 kb to more than 100 kb [13,61]. In this study, different approaches have been applied to identify and characterize *A. baumannii* sequenced strains: PFGE with an S1 endonuclease digestion, PCR detection with specific *A. baumannii* plasmids primers, and PLACNETw software as bioinformatic analysis.

Regarding the PFGE profile, as far as we know, this was never used before for *Acinetobacter* plasmid detection, but for patterns of chromosomic DNA [28]. For PCR detection, specific primers for *A. baumannii* plasmids designed by Bertini et al. were used, in which the authors grouped the resistance plasmid by replicase homology (19 groups). However, based on subsequent results, these authors’ PBRT scheme might be reviewed and new homology groups added, due to the increasing number of this bacterium plasmid sequence [13,53]. PLACNETw was used not only to detect, but also to identify the plasmid type, which is limited by the annotated and deposited genomes [62].

Taking the results of the three approaches together, we obtained common predictions in most strains, highlighting the importance of using different validating methods. In the ATCC^®^19606^TM^ strain, plasmid prediction was positive through two methods and the PLACNETw putative plasmid had enough identity to be considered as a *pMAC* [63], which is classified in Bertini GR8. Small-sized plasmids could be the reason for not obtaining a PFGE prediction (Figure 4B). In the HUMV1319 strain, the PLACNETw server identified a putative *pMMCU3* plasmid of 8.9 kb, which is included in the Bertini classification as GR2. The same results were obtained for one small HUMV2471 plasmid (8.9 kb), and a high identity of the 6 kb plasmid with the *pRAY* structure [13]. The *pMMCU3* sequence similarities were confirmed through a Clustal W [64] alignment, being the strains that harbored the *bla_OXA-24/40_* gene typically carried by this mobile structure [38]. In HUMV2471, two plasmids of 118.39 and 69.2 kb were also identified by PLACNETw and positively identified with similar sizes in the PFGE gel, but not enough identity was obtained in PLACNETw to classify them correctly, while the biggest one presented some similarity with *pABUH4-111* and *pABTJ2* plasmids [59]. In HUMV2790, a band between 97 and 145.5 kb with a PFGE analysis showed the presence of a big plasmid, which was also detected with PLACNETw as a 105.1 kb structure identified as *pABUH5-114*, recently described for the first time as a large *A. baumannii* plasmid that carries phage-related genes [53]. The 11.55 kb small one predicted with the bioinformatic tools as a putative *pMMCU3* plasmid could correlate with the Bertini GR2 positive classification. Furthermore, in HUMV3743 through PFGE identification, two plasmids of 97–145.5 kb and 48.5–97 kb were detected in concordance with the PLACNETw putative plasmid sizes predicted (107 and 78 kb, respectively), and identified as *pABUH5*-*114* and *pABUH1*-*74*, respectively, included in *pACICU2*-like plasmids which belong to Bertini GR6 [15,53].

Therefore, putting this all together, both traditional microbiology and genomic approaches could be useful to decipher the nosocomial pathogens’ behaviors when more routine methods are not enough. In addition, using genomic tools for non-enterobacteria would improve our knowledge of human pathogen bacteria even it is usually a bigger challenge due to the lack of databases for them.

## 4. Materials and Methods

### 4.1. Bacterial Strains

Four *A. baumannii* clinical isolates were included in this work. All isolates were obtained from different patients (standard service of routine) at the Hospital Universitario Marqués de Valdecilla (HUMV) from Santander (Spain), whose phenotypes were previously described [22]. HUMV1319 and HUMV3743 were isolated from wound exudate, HUMV2471 from sputum, and HUMV2790 from a skin ulcer. Reference *strain A. baumannii* ATCC^®^19606^TM^ [19] was also included. *A. baumannii* strains were routinely cultured on blood agar (BA) plates, or Luria broth (LB) at 37 °C, and stock cultures were frozen at −80 °C with 20% (vol/vol) glycerol.

### 4.2. Antibiotic Susceptibility Assays

Antibiotic susceptibility was tested by microdilution following CLSI guidelines [25]. Seven compounds belonging to 6 of the main antibiotic families were tested: ampicillin, meropenem, colistin, ciprofloxacin, erythromycin, gentamicin, and tetracycline. EUCAST 2019 breakpoints for *Acinetobacter* spp. [24] were used to classify the isolates as resistant or susceptible. Briefly, bacterial suspension was adjusted to OD_620_ = 0.05, which equals 0.5 McFarland units. Antimicrobials were tested at the following ranges: ampicillin 1–64 mg/L, meropenem 0.25–6 mg/L, colistin 0.125–8 mg/L, ciprofloxacin 0.06–4 mg/L, erythromycin 2–128 mg/L, gentamicin 0.5–32 mg/L, and tetracycline 0.5–32 mg/L.

### 4.3. DNA Isolation and Sequencing

The total genomic sample of A. baumannii clinical strains and the ATCC^®^19606^TM^ reference one was extracted and purified using the GeneJET genomic DNA (gDNA) isolation kit (Thermo Scientific, Carlsbad, CA, USA) after growth on LB for 24 h at 37 °C. The gDNA was submitted to Fisabio (Valencia, Spain) for Illumina (MiSeq 2 × 300 bp) sequencing. DNA libraries were generated following the Nextera XT Illumina protocol (Nextera XT library prep kit [catalog number FC-131-1024]). Next, 0.2 ng/μL of purified gDNA was used to initiate the protocol. The multiplexing step was performed using the Nextera XT index kit (catalog number FC-131-1096). The libraries were sequenced using a 2 × 300 bp paired-end run (MiSeq v3 reagent kit [catalog number MS-102-3003] on a MiSeq sequencer according to the manufacturer’s instructions [Illumina, Valencia, Spain]).

### 4.4. Genomic Assembly and Annotation

The whole-genome sequence was assembled using Unicycler v0.3.0.b with both paired and unpaired reads [65].

Annotation of the genome sequence was performed with the Rapid Annotations using Subsystems Technology (RAST) server, as well as Prokka 1.13 to identify the number of coding sequences (CDS), subsystem feature counts, and RNA identification [32,50]. The genotyping was performed using the PubMLST database [66].

Gene clusters related to the secondary metabolite biosynthetic were detected using antiSMASH (bacterial version) [67]. For antimicrobial resistance genes, a preliminary analysis using Prokka 1.13 [32] and ABRicate 0.8 software (https://www.github.com/tseemann/abricate, accessed on: 1 June 2018) (Resfinder 3.2 database) [68], as well as CARD database [30] for efflux pumps encoding genes were used. BLAST alignment with the tblastn option (https://blast.ncbi.nlm.nih.gov/, accessed on: 1 June 2018) was used to identify specific genes. The software MEGA7 was used for point mutation identification [31], as well as the PointFinder database from ABRicate software.

### 4.5. Plasmid Prediction and Identifications

The PLACNETw server was used to predict feasible plasmids in Illumina sequenced genomes [62]. Identification using the Bertini et al. [15] approach for PCR-based replicon typing (PBRT) with specific *A. baumannii* resistance plasmid primers was also used to detect and characterize the positive harboring strains. Plasmid presence identification by Pulsed-field Gel Electrophoresis (PFGE) digestion was performed with a CHEF-DR^®^ III system (Bio-Rad, Hercules, CA, USA) as following. Bacteria were grown in LB overnight (O/N) with shaking (175 rpm), and 500 µL of bacterial suspension was mixed with the same volume of 2% LM agarose in TE buffer (10 mM Tris, 1 mM EDTA) for making plugs. Then, 1 mL of lysis solution with 0.5 mg/mL of lysozyme was added and incubated O/N at 37 °C with shaking. After, an equal volume of 0.5 mg/mL of proteinase K in ES (EDTA 0.5 M, sarcosyl 1%) buffer was added and incubated during 16–20 h at 56 °C in static. The plugs were washed six times with pre-warmed TE buffer and then digested with 20 U of S1 restriction enzyme for 30 min at 37 °C. Electrophoresis was performed in a 1% agarose gel at 6 V/cm and 14 °C with 0.5× TBE buffer. Pulse times ramped from 1 to 15 s for 6 h and 15 to 35 s for 16 h. Low range PFGE marker (New England Biolabs, Ipswich, MA, USA) was used as the molecular size marker.

### 4.6. Strain Comparison and Pan-Genome Analysis

ANI [57] and DDH [58] values were calculated for each strain to determine evolutionary distances and to identify the species, using AYE [17] as a reference strain.

For pan-genome comparison, PanOCT software [55] was used to cluster orthologous proteins, and the EggNOG v4.0 database [69,70] to annotate their functional categories. InteractiVenn was used for diagram illustration [56].

## Figures and Tables

**Table 1:** Draft *A. baumannii* genomes features according to RAST and Prokka annotation. In ATCC19606 and HUMV2471, one repeat region was also identified in each strain. *CDS*, coding sequences.

		Strains				
		ATCC19606	HUMV1319	HUMV2471	HUMV2790	HUMV3743
Contigs		96	110	108	118	176
Size (bp)		3,914,294	3,916,888	3,810,872	3,935,804	4,006,761
% GC		39.1	39	38.9	39	39
Subsystems		462	451	453	453	451
CDS	RAST	3689	3703	3556	3706	3882
	Prokka	3667	3680	3542	3689	3886
tRNA		64	63	64	62	63
mRNA		1	1	1	1	1
rRNA		3	3	3	3	2
MLST	Oxford	ST921	ST106		ST350	ST218
	Pasteur	ST52	ST3	ST49	ST2	ST2

**Table 2:** Susceptibility of *A. baumannii* strains against seven antimicrobial agents. Isolates were classified as resistant according to EUCAST breakpoints [24]. For the resistance profile: *COL (C)* is colistin; *MER (M)* is meropenem; *AMP (A)* is ampicillin; *GEN (G)* is gentamicin; *CIP (P)* is ciprofloxacin; *TET (T)* is tetracycline; and *ERY (E)* is erythromycin. *MIC* is minimum inhibitory concentration; *MIC_50_* is MIC that inhibits 50% of the isolates; *MIC_90_* is MIC that inhibits 90% of the isolates. * No specific value for *Acinetobacter*, used general no species-specific breakpoints. ** CLSI breakpoints used [25].

		COL	MER	AMP *	GEN	CIP	TET **	ERY	Resistance Profile
**MIC**	**ATCC19606**	2	4	>64	4	>0.25	1	>128	MAE
	**HUMV1319**	2	>16	>64	>128	>4	8	>128	MAGPTE
	**HUMV2471**	2	>16	>64	>128	>4	16	32	MAGPTE
	**HUMV2790**	8	4	>64	8	>4	>32	>128	CMAGPTE
	**HUMV3743**	4	>16	>64	>128	>4	>32	64	CMAGPTE
	**MIC_50_**	2	>16	>64	>32	>4	16	>128	
	**MIC_90_**	>8	>16	>64	>32	>4	>32	>128	
	**Range (mg/L)**	0.125–8	0.25–16	1–64	0.5–32	0.06–4	0.5–32	2–128	

**Table 3:** Antimicrobial resistance genes identified with Prokka and CARD databases.

STRAINS	ANTIMICROBIAL CLASSES RESISTANCE GENES			
	AMINOGLYCOSIDES	β-LACTAM		SULFONAMIDES
		*ampC*	OXA	
**ATCC19606**	*ant(3′’)-IIa*	ADC-2	OXA-51 (98)	*sul*
			OXA-51 (386)	
**HUMV1319**	*aac(6’)-Ib’*	ADC-7	OXA-24 OXA-51 (71) OXA-51 (385)	*sul*
	*ant(2’’)-Ia*			
	*ant(3’’)-IIa*			
	*aph(3’)-VI*			
	*aph(3’)-VIa*			
**HUMV2471**	*ant(2’’)-Ia* *ant(3’’)-IIa*	ADC-39	OXA-24	
			OXA-51 (98)	
			OXA-51 (386)	
**HUMV2790**	*ant(3’’)-IIa* *aph(3’’)-Ib* *aph(6)-Id*	ADC-25	OXA-51 (66) OXA-51 (109)	*sul*
**HUMV3743**	*aac(3)-IIa* *ant(3’’)-IIa* *aph(3’’)-Ib* *aph(3’)-VIa* *aph(6)-Id*	ADC-25	OXA-51 (66) OXA-51 (109)	

## Data Availability

HUMV1319, HUMV2471, HUMV2790, and HUMV3743 whole-genome shotgun projects have been deposited at DDBJ/ENA/GenBank under the accession numbers SRR13361577, SRR13361576, SRR13361575, and SRR8264720, respectively; included in the BioProject PRJNA507520.

## Supplementary Materials

The following are available online at https://www.mdpi.com/article/10.3390/antibiotics10070753/s1, Figure S1: Plasmid prediction with PLACNET of 5 *A. baumannii* strains; Table S1: List of virulence genes of 5 *A. baumannii* strains.
