# Supplementary material for: Antimicrobial Resistance Determinants in Genomes and Plasmids from Acinetobacter baumannii Clinical Isolates
Source: Antibiotics (Basel). 2021 Jun 22;10(7):753. doi: 10.3390/antibiotics10070753 (PMC8300758; doi:10.3390/antibiotics10070753)
Supplement: Supplementary file 1 [file antibiotics-10-00753-s001.zip › Figure S1.pdf]

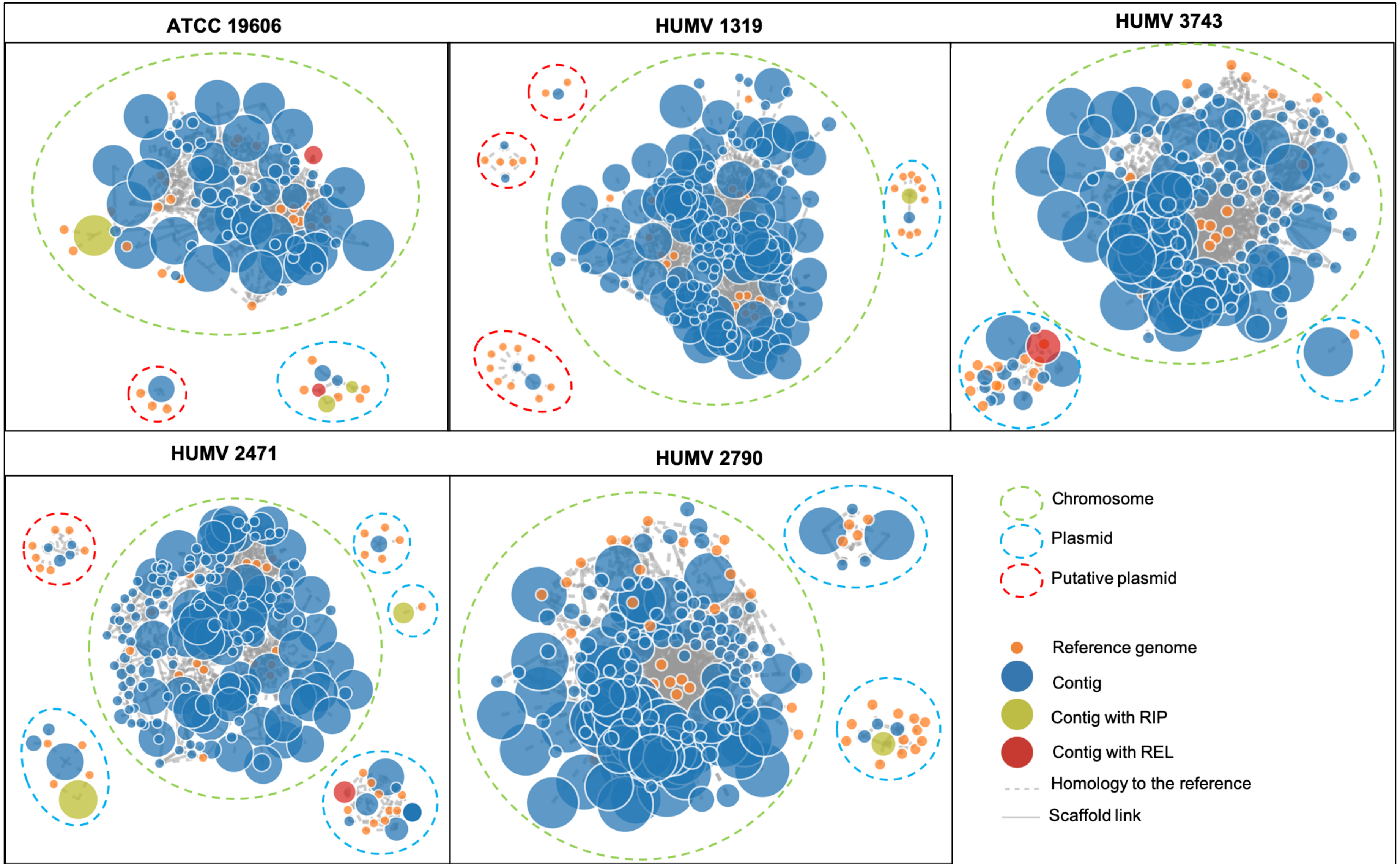

Figure S1. Plasmid prediction with PLACNET of 5 *A. baumannii* strains. Each circle (blue, red and green) represents one contig and its dimension is proportional to its size. Dotted green lines round contigs that conform the genome; blue lines: contigs that conform predicted plasmid with high similarity to reference ones; red lines putative plasmids structures that have no enough size and/or similarity to be described as one. In ATCC®19606™ two putative plasmids of 11 and 15 kb were predicted; in HUMV1319 an 8.90 kb plasmid and three smaller accessory structures from 1 to 7 kb; in HUMV2471 four plasmids of 118.39, 69.2 kb, 9.85 kb and 6 kb; in HUMV2790 a 105.1 kb and 11.55 kb plasmids; and in HUMV3743 two of over 107 kb and 78.73 kb. *RIP*, replication initiator proteins; *REL*, relaxases.
